# Treatment response and clinical features of snakebite envenomation in Alborz province, Iran: A cross‐sectional study

**DOI:** 10.1002/hsr2.70135

**Published:** 2024-10-15

**Authors:** Hoorvash Farajidana, Seyedarad Mosalamiaghili, Kasra Assadian, Soodeh Jahangiri, Maryam Masumzadegan, Farangis Sadeghi, Lida shojaei Arani

**Affiliations:** ^1^ Emergency Department, Kosar Hospital Poison Center Alborz University of Medical Science Karaj Iran; ^2^ Burn and Wound Healing Research Center Shiraz University of Medical Sciences Shiraz Iran; ^3^ Student Research Committee Shiraz University of Medical Sceinces Shiraz Iran; ^4^ Endocrine Research Center, Institute of Endocrinology and Metabolism Iran University of Medical Sciences Tehran Iran; ^5^ Jahromi Research Committee Alborz University of Medical Sciences Karaj Iran; ^6^ Clinical Development Research Unit, Sayad Shirazi Hospital Golestan University of Medical Sciences Gorgan Iran

**Keywords:** antivenom therapy, clinical outcomes, envenomation severity, snakebite

## Abstract

**Background:**

Snakebite envenomation is a significant yet neglected public health burden. Our aim was to investigate the clinical and demographic factors of snakebite envenomation, as well as the factors associated with its severity and response to treatment, in Alborz province, northern Iran.

**Methods:**

In this cross‐sectional study, we included 50 patients diagnosed with snakebite envenomation, referring to the Poison Control Centre of Alborz University of Medical Sciences. The presenting signs and symptoms, demographic data, treatment dose, response to treatment, complications, laboratory findings, and snakebite severity scale (SSS) were collected.

**Results:**

Forty‐six patients (92%) were men, the total mean age was 31.7 ± 12.06 years, and mean SSS was 6.54 ± 3.39. The patients were admitted for a median of 2 days, and 11 patients needed ICU admissions. The majority of snakebites were in upper extremities (60%) and they mainly occurred in summer (56%). SSS was significantly associated with response to treatment, ICU admission, gastrointestinal adverse events, thrombocytopenia, and length of stay. Similarly, response to treatment was significantly related to the history of snakebite, ICU admission, gastrointestinal adverse events, thrombocytopenia, length of stay, and SSS.

**Conclusion:**

Gastrointestinal symptoms, higher severity scores, and longer hospital stays were associated with poor treatment response. Importantly, no mortality was observed in this cohort. Further research is needed to confirm these findings and optimize treatment strategies for snakebite management.

## 1 INTRODUCTION

Snakebite envenoming is a significant and neglected public health concern in many parts of the world^1^; the approximate prevalence of snakebite is 4–5 million yearly, of which 1.8–2.7 million cases are envenomed.^2^ In Iran Snake envenomation reports reaches approximately 4500–7000 snakebite cases annually^3^ with 3–9 deaths reported per year.^4^ Snakebite envenoming poses a considerable threat to human health, with potential local and systemic consequences if left untreated or mismanaged.^5^ Understanding the clinical factors associated with snakebites and evaluating the response to treatment is crucial for improving patient outcomes and guiding medical interventions.^6^

**Figure 1:**
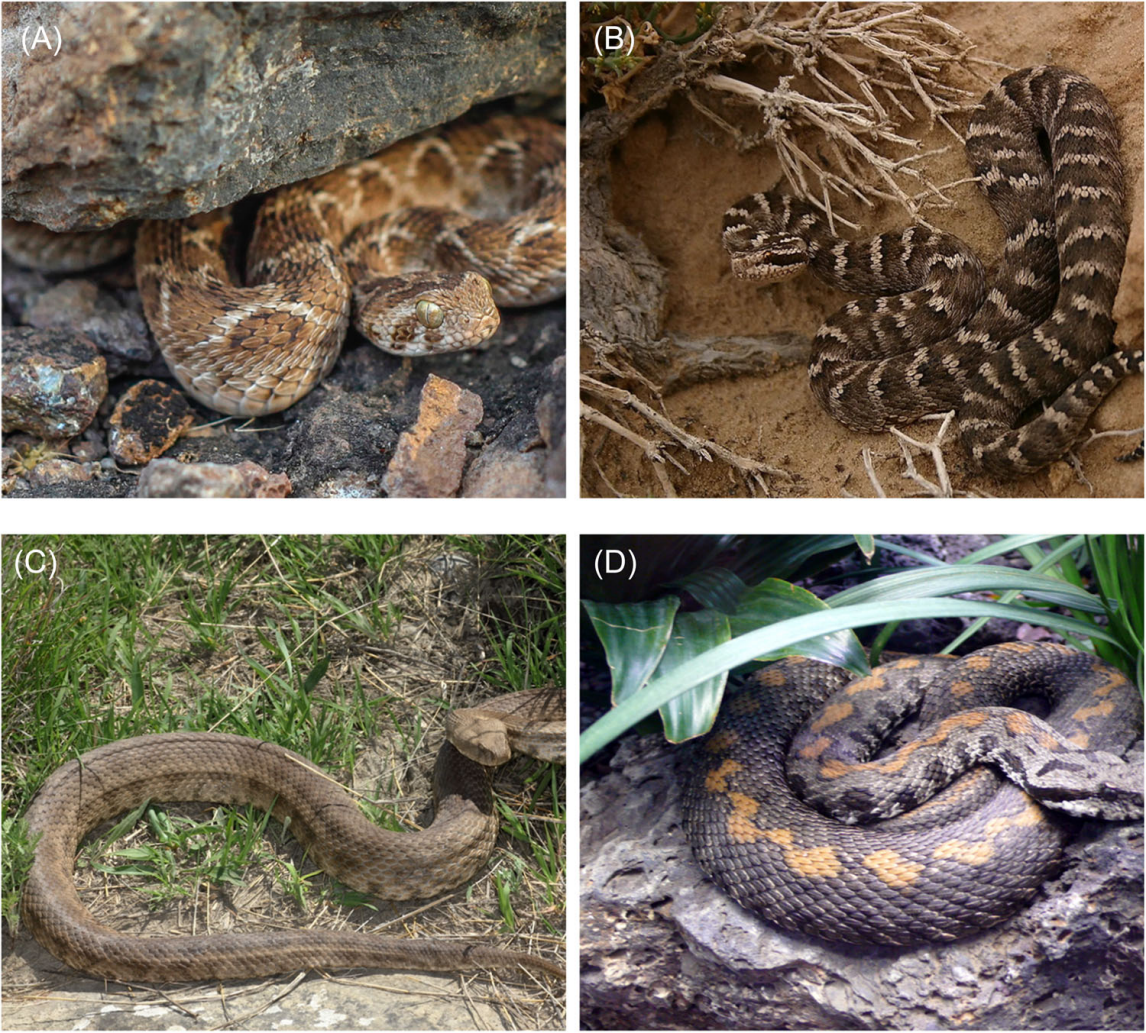
Venomous snakes commonly encountered in the region: (A) *Echis carinatus*, (B) *Gloydius intermedius*, (C) Macrovipera lebetinus, and (D) Montivipera raddei albicornuta.

Snakebite incidents in Iran pose unique challenges due to the diverse snake species found in the area, including venomous ones such as the *Levantine viper (Macrovipera lebetina)* and the saw‐scaled viper (*Echis carinatus*) (Figure 1).^4^, ^7^ The national guidelines recommend using Razi polyvalent antivenom (equine F(ab')2 antivenom, Razi Vaccine and Serum Research Institute, Iran) along with supportive treatment for managing snakebites, with an initial dose of 5–10 vials administered intravenously. Supportive treatment includes administering medications such as epinephrine and antihistamines, managing airways, providing blood products, and initiating IV hydration.^3^

**Figure 2:**
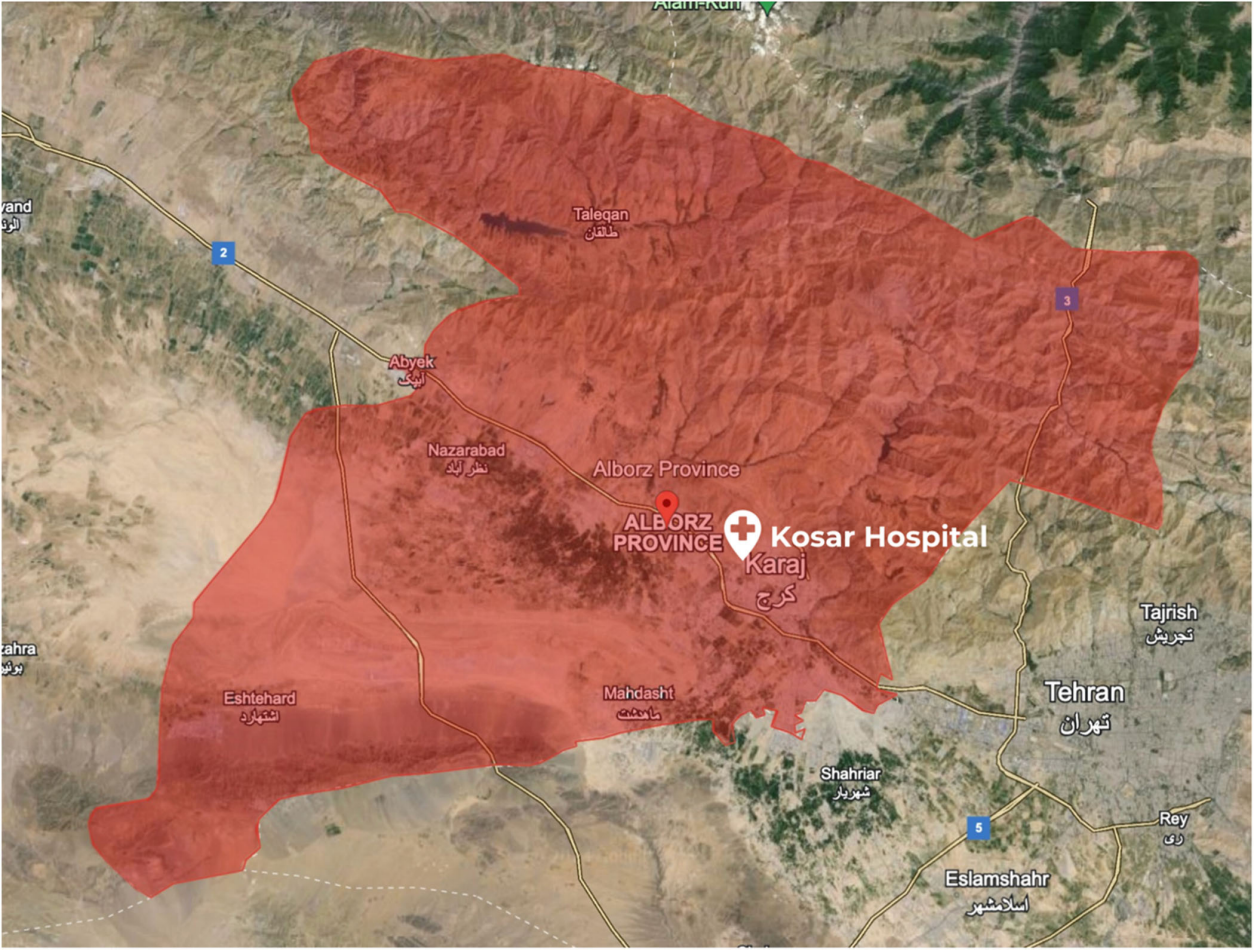
Map showing the region of Iran were patients have referred to the hospital. Map data © 2023 Google Imagery © Airbus.

This research investigates the clinical profiles and outcomes of patients envenomed by four venomous snake species (*Echis carinatus*, *Gloydius intermedius*, *Macrovipera lebetinus*, and *Montivipera raddei albicornuta*) (Figure 1) commonly found in the region (Figure 2), aiming to provide a comprehensive understanding of the regional snakebite envenomation landscape. Given the geographical variations in snake species and venom composition, it is necessary to conduct region‐specific research on snake envenomation. Therefore, the aim of this study was to gain insight into the demographics, presentation, and severity of snakebite envenomation cases at a referral hospital in the centeral Iran (Alborz province), representing a regional perspective on the issue. Additionally, we investigated factors associated with the severity of snakebites and their response to treatment, with the goal of enhancing current management protocols.

## 2 METHODS

### 2.1 Subjects and study design

This cross‐sectional study was conducted from March 2020 to February 2022 at Kowsar Hospital, Poison Control Centre of Alborz University of Medical Sciences, Iran. All patients referring with a snakebite envenomation within 24 h were selected for inclusion in the study and their data was collected retrospectively. The exclusion criteria were as follows: patients with suspected snakebite, patients presenting >24 h of snakebite, those who did not receive antivenom, and incomplete data.^8^

The current study was carried out under the Helsinki Declaration and approved by the ethics committee of Alborz University of Medical Sciences (Approval number: IR.ABZUMS.REC.1401.135).

### 2.2 Data acquisition and snakebite severity score

A pre‐defined questionnaire was filled out to using the hospital records to obtain the patients' data including age, gender, ethnicity, history of previous snakebites, presenting signs and symptoms, site of snakebite, length of stay as the number of days the patients spent in the hospital, type of admission (i.e., ward or intensive care unit), antivenom dose calculated as number of received vials of antivenom, and laboratory findings. Coagulation abnormalities in lab data were defined as prolonged plasmin time (PT) and International normalized ratio (INR), partial thromboplastin time (PTT), and bleeding time (BT). Response to treatment was indicated as adequate when ≤30 vials of antivenoms were used, and poor when >30 vials of antivenom were used. The snakebite severity score (SSS) was calculated for each patient.^9^ SSS is a clinical tool for assessing the severity of snakebite, ranging from 0 to 20. Participants were categorized into the following groups based on SSS: mild (score of 0–3), moderate (score of 4–7), and severe (score of 8–20).

### 2.3 Statistical analysis

To comply with best reporting standards, recommendations of Assel^10^ were followed. Statistical analyses were conducted using IBM SPSS Statistics for Windows (Version 26.0 IBM Corp. Armonk, NY). Categorical variables are presented as numbers (%), and continuous variables are presented as mean ± standard deviation (SD) or median with interquartile ranges (IQR). To assess the association between variables, the following tests were used: chi‐squared or Fisher's exact test, independent samples T test or Mann‐Whitney U test, one‐way analysis of variances (ANOVA) or Kruskal‐Wallis test, and Kendall's Tau and Spearman's rank correlation tests. A two‐sided *p*‐value < 0.05 was considered significant for all tests.

## 3 RESULTS

Fifty patients were included in the study, 46 (92%) were male and the mean age was 31.7 ± 12.06 years. The mean SSS score was 6.54 ± 3.39 and ranged from 1 to 15, and a median number of 25 antivenoms were used. Median length of stay was 2 days, and 11 patients (22%) required ICU admission during their hospitalization. All participants were local residents. Response to treatment was adequate in 33 (66%) of patients. The ethnicities were as follows: 23 (46%) patients were Fars (Persian), 19 (38%) were Afghan, and the rest were Turkic (Table 1).

**Table 1:** Characteristics of the study population (*n* = 50) including demographic data, clinical presentations, adverse events, and laboratory findings of snakebite patients.

Variable	Description
Age, *median (IQR)*	28.5 (15.25)
SSS, *mean ± SD*	6.54 ± 3.39
Antivenom dose, *median (IQR)*	25 (15–35)
Length of stay, *median (IQR)*	2 (2–3)
ICU admission, *n (%)*	11 (22)
Duration of ICU admission, *median (IQR)*	2 (1–2)
Signs, *n (%)*	
Edema	47 (94)
Ecchymosis	43 (86)
Blister	35 (70)
Adverse events, *n (%)*	
GI	9 (18)
Respiratory distress	4 (8)
Cardiovascular	2 (4)
Neurologic	3 (6)
Lab data, *n (%)*	
Thrombocytopenia	8 (16)
High serum Cr	5 (10)
K disturbance	6 (12)
Na disturbance	3 (6)
Ca disturbance	4 (8)
Abnormal PT	11 (22)
Abnormal PTT	5 (10)
Abnormal INR	10 (20)
Abnormal BT	3 (6)

Abbreviations: BT, bleeding time; Cr, creatinine; GI, gastrointestinal; PT, prothrombin time; PTT, partial thromboplastin time; SSS, snakebite severity score.

Envenomation occurred by the following snakes: *Echis carinatus, Vipera albicornuta, Vipera lebetina obtusa*, and *Agkistrodon halys*. The bite occurred most commonly in summer (56%) and majorly in upper extremities (60%). Edema was the most abundant presenting sign, occurring in 94% of patients. Subsequently, ecchymosis and blister were present in 86% and 70% of patients, respectively.

Gastrointestinal disturbance occurred in nine (18%) participants and was the most common complication. Four patients (8%) developed respiratory distress, three (6%) had neurologic complications, and cardiovascular adverse events were present in two (4%).

There were 16 (32%) cases with coagulopathy; prolonged PT, PTT, and BT were present in 11 (22%), five (10%), and three (6%) patients, respectively. Also, 16% of cases had thrombocytopenia.

According to SSS, the snakebite was mild in nine (18%) patients, moderate in 30 (60%) patients, and severe in 11 (22%) patients. Response to treatment, ICU admission, Gastrointestinal adverse events, thrombocytopenia, and length of stay were significantly associated with the SSS group (Tables 2, 3). Also, History of snakebite, ICU admission, Gastrointestinal adverse events, thrombocytopenia, length of stay, and SSS were significantly related to response to treatment.

**Table 2:** The association between categorical variables and patient groups based on snakebite severity score (SSS) and response to treatment.

Variable		SSS					*P*‐value	Response to treatment		*P*‐value
		Mild *n* = 9		Moderate *n* = 24		Severe *n* = 17		Adequate *n* = 33	Poor *n* = 17	
Gender										
Female		‐		3 (75)		1 (25)	0.654	3	1	>0.99
Male		9 (19.6)		21 (45.7)		16 (34.8)		30	16	
Site										
Upper extremities		7 (23.3)		13 (43.3)		10 (33.3)	0.517	20 (66.7)	10 (33.3)	>0.99
Lower extremities		2 (10)		11 (55)		7 (35)		13 (65)	7 (35)	
Ethnicity										
Fars		3 (13)		13 (56.5)		7 (30.4)	0.135	16 (69.6)	7 (30.4)	0.210
Turkic		4 (50)		3 (37.5)		1 (12.5)		7 (87.5)	1 (12.5)	
Afghan		2 (10.5)		8 (42.1)		9 (47.4)		10 (52.6)	9 (47.4)	
History of snakebite										
Yes		‐		‐		3	0.07	‐	3	0.035
No		9 (19.1)		24 (51.1)		14 (29.8)		33 (70.2)	14 (29.8)	
Signs										
Edema		9 (19.1)		22 (46.8)		16 (34)	>0.99	31 (66)	16 (34)	>0.99
Blister		6 (17.1)		16 (45.7)		13 (37.1)	0.779	22 (62.9)	13 (37.1)	0.533
Ecchymosis		8 (18.6)		20 (46.5)		15 (34.9)	>0.99	28 (65.1)	15 (34.9)	>0.99
Adverse events										
Respiratory distress		‐		1 (25)		3 (75)	0.298	1 (25)	3 (75)	0.108
Cardiovascular		1		‐		1	0.265	1	1	‐
Gastrointestinal		‐		2 (25)		6 (75)	0.031	2 (25)	6 (75)	0.013
Neurologic		1		1		‐	0.442	2	‐	0.542
ICU admission										
Yes		‐		3 (27.3)		8 (72.7)	0.007	3 (27.3%)	8 (72.7%)	0.004
No		9 (23.1)		21 (53.8)		9 (23.1)		30 (76.9%)	9 (23.1%)	
Response to treatment										
Poor		‐		‐		17	<0.0001	‐	‐	‐
Adequate		9 (27.3)		24 (72.7)		‐				
Laboratory findings										
Thrombocytopenia		1		1		6	0.019	2	6	0.013
Cr		‐		2		3	0.564	2	3	0.321
K		2		1		3	0.208	3	3	0.396
Na		1		‐		2	0.177	1	2	0.264
Ca		‐		1		3	0.298	1	3	0.108
Coagulopathy		1		7		8	0.172	8	8	0.121

*Note*: Data are presented as *n* (%) for each category. *P*‐values are calculated using Chi‐square or Fisher's exact test as appropriate. *p* < 0.05 is considered statistically significant.

**Table 3:** The association between continuous variables and patient groups based on snakebite severity score (SSS) and response to treatment.

Variable	SSS			*P*‐value	Response to treatment		*P*‐value
	Mild	Moderate	Severe		Adequate	Poor	
Age	32 (14.5)	28 (12)	34 (26.5)	0.965	28 (11)	34 (26.5)	0.907
Antivenom dose	10 (0)	20 (8.75)	35 (2.5)	<0.0001	‐	‐	‐
Length of stay	2 (0.5)	2 (1)	3 (0.5)	<0.0001	2 (0.5)	3 (0.5)	<0.0001
SSS	‐	‐	‐	‐	5 (3)	10 (3)	<0.0001

*Note*: Data are presented as median (Interquartile range). *P*‐values are calculated using Kruskal‐Wallis test for SSS groups and Mann‐Whitney U test for response to treatment groups. *p* < 0.05 is considered statistically significant.

Abbreviation: SSS, snakebite severity score.

The correlation analysis revealed a significant association between SSS and antivenom dose, SSS and length of stay, and antivenom dose and length of stay (Table 4).

**Table 4:** Matrix of correlations between age, antivenom dose, length of stay, and snakebite severity score (SSS).

Variables	Age	Antivenom dose	Length of stay	SSS
Age	‐	−0.062	−0.032	−0.088
Antivenom dose	−0.062	‐	0.679^a^	0.899^a^
Length of stay	−0.032	0.679^a^	‐	0.648^a^
SSS	−0.088	0.899^a^	0.648^a^	‐

*Note*: Values represent Spearman's correlation coefficients. Asterisks (*) indicate correlations significant at the 0.01 level (2‐tailed). *p* < 0.05 is considered statistically significant.

^a^ Correlation is significant at the 0.01 level (two‐tailed).

## 4 DISCUSSION

This study aimed to investigate prognostic factors in patients with snakebite complaints admitted to the Alborz Poison Center between March 2020 to February 2022. The results of this study provide valuable insights into the demographic characteristics, clinical manifestations, laboratory findings, and their associations with snakebite severity scores and prognosis.

The demographic characteristics of the patients included in this study revealed a higher prevalence of snakebites among males, consistent with most previous studies.^11^, ^12^, ^13^ The mean age of the patients was 31.7 years, reflecting a relatively young population affected by snakebites. Previous studies also reported similar gender and age distribution between the patients.^14^, ^15^ These findings can be attributed to the fact that males in the younger age group tend to be more active and engaged in outdoor activities.

Regarding the site of snakebite, the upper extremities were more commonly affected (60%) compared to the lower extremities (40%). Previous studies have yielded mixed results, with one study reporting higher rates of snakebites on upper limbs,^16^ while others report higher incidence on lower limbs.^11^, ^17^, ^18^ The behavior and ecology of snakes, whether arboreal or terrestrial, play a significant role in determining the site of snakebites. However, further research may be necessary to fully understand this relationship.

Clinical manifestations following snakebite varied among the patients. Edema, ecchymosis, and blisters were the most common initial clinical signs observed, highlighting the local tissue effects of snake envenomation. These symptoms were commonly seen in previous studies as well.^19^, ^20^ The severity of local inflammation varies depending on the type of snake venom, but it is thought to be caused by myonecrosis, inflammatory cytokines, and increased vascular permeability.^1^

Gastrointestinal symptoms were the most frequent systemic manifestations, followed by respiratory distress. These findings align with previous studies reporting the systemic effects of snake venom on multiple organ systems.^21^, ^22^ Other studies also mentioned systemic bleeding such as hematuria, hemoptysis, and symptoms of disseminated intravascular coagulation (DIC).^23^ Neurologic symptoms, fasciculation, and cardiovascular involvement have also been reported, although less frequently.^19^, ^24^

Laboratory findings showed several abnormalities associated with snake envenomation. Prolonged PT and increased INR were the most common laboratory abnormalities, reflecting disseminated intravascular coagulation. Thrombocytopenia, potassium imbalances, and elevated creatinine levels were also commonly observed. These findings are consistent with the hematological and renal disturbances observed in previous studies.^25^, ^26^, ^27^, ^28^ Other laboratory abnormalities, such as prolonged PTT, calcium imbalance, sodium imbalance, and elevated bilirubin levels, were less prevalent in this study cohort. Other studies have also mentioned neutrophil‐dominant leukocytosis, increased D‐dimer levels, and elevated fibrin degradation product.^1^, ^29^ The most common electrolyte abnormalities following snakebite include hyponatremia, hypocalcemia, and hypokalemia which are believed to result from renal tubular loss.^25^, ^27^

In the context of the venom proteomes of the species in our study (*Echis carinatus*, *Macrovipera lebetina*, etc.), local tissue damage such as edema and systemic complications like thrombocytopenia are likely due to enzymes like metalloproteinases and disintegrin.^30^, ^31^ Specifically, *Echis carinatus* venom contains components like EC1.5(a), a metalloproteinase with strong pro‐coagulant and hemorrhagic activities, and EC5.1(b), which has anti‐coagulant and antiplatelet properties.^32^ These venom proteins may explain the coagulopathy and thrombocytopenia observed in our patients. Further research should explore these components and their clinical implications.

Various methods have been proposed to classify the severity of snakebite incidents; however, a significant drawback of many existing approaches is their subjectivity. One widely recognized scoring system is the SSS, which offers an objective approach to assess the severity of snakebite envenomation.^9^ The SSS incorporates multiple components such as local symptoms, systemic manifestations, and laboratory parameters. Each component is assigned a numerical score, and the cumulative score (0–20) provides a quantitative measure of the severity of snakebite envenomation. Scores of 0–3 indicate mild cases, 4–7 represent moderate cases, and 8–20 indicate severe cases.^9^

The majority of our patients fell into the moderate severity category. The severity score did not show any significant association with demographic factors. Among clinical manifestations, gastrointestinal disturbances were the only symptoms found to have a significant association with higher severity scores. No abnormal laboratory data was significantly associated with the severity score. As expected, a higher severity score was positively associated with ICU admission, poor response to treatment, number of anti‐venoms used, and length of hospital stay. These findings highlight the critical role of timely treatment of the complications associated with severe snake envenomation.

In our study, patients were classified into two groups based on the number of antivenom vials used (good response for below 30, and poor response for more than 30). History of previous snake envenomation, presence of gastrointestinal symptoms, thrombocytopenia, ICU admission, higher severity score, and longer duration of hospitalization were significantly associated with poor response to treatment. Therefore, in patients with these characteristics, snakebite should be considered severe and treatment and supportive measures should be taken seriously. Yin et al. demonstrated that thrombocytopenia, coagulopathy, spontaneous bleeding, and neurologic symptoms are the most important indicators of poor patient condition.^33^

The treatment of snakebite requires a multidimensional approach to manage venom‐induced systemic effects and local tissue damage.^34^ The basis of treatment is the administration of specific antivenom (also known as antivenin or antivenom serum) to neutralize the venom's toxic components. Antivenom therapy has been shown to significantly improve patient outcomes by reducing both morbidity and mortality.^35^, ^36^ Timely initiation of antivenom treatment is crucial to prevent the progression of systemic envenomation. Supportive care measures, including maintaining vital organ functions, managing coagulopathies, and addressing hemodynamic instability, play a vital role in the management of severe snakebites. In cases of severe local tissue damage, surgical interventions such as debridement, fasciotomy, or skin grafting may be required to prevent long‐term functional impairment and promote wound healing.^37^ Furthermore, adjunctive therapies such as pain management, tetanus prophylaxis, and antibiotics to prevent secondary infections are commonly employed as part of the comprehensive treatment strategy.^38^

Our study revealed no mortality caused by snakebite among the patients. Previous studies have reported mortality rates ranging from 3% to 58% in different centers.^39^, ^40^ This disparity could be due to different snake species, venom composition, accessibility to medical facilities, treatment protocols, and healthcare provider expertize. In this regard, Sharma et al. demonstrated that hospitalization of snakebite patients in specialized facilities equipped with ventilators, sufficient antivenom, and dialysis machines is significantly associated with a reduction in mortality rates.^41^ Therefore, higher rates of morbidity and mortality have been reported in rural healthcare centers. It is also worth mentioning that mortality rates tend to be higher in children due to their increased toxin absorption per kilogram of body weight compared to adults.^42^

Our study did not collect specific data regarding the cost and long‐term outcomes of antivenom treatment. Salim et al., in their analysis of snakebite cases in private hospitals in India, highlighted the importance of cost‐effectiveness analysis in improving treatment accessibility. They demonstrated that 80% of patients could be treated for around USD 1200 or less, with factors such as snake type, complications, and delays in treatment driving costs.^43^ In regions like Iran, the absence of such data limits our understanding of the broader socioeconomic impact of snakebite envenomation. We recommend that future studies assess the economic impact of snakebite management in Iran, with a specific focus on the availability and cost of antivenom.

The present study has several limitations that should be acknowledged and taken into consideration. First, our sample size was relatively small, which may limit the generalizability of the results to the population of snakebite patients. A larger sample size would improve the statistical power and provide a more representative picture of prognostic factors in snakebite cases. Another limitation is the lack of specific identification of snake species in the study. The venom composition can vary significantly among different snake species, leading to variations in clinical manifestations and treatment responses. Without accurate species identification, it is challenging to determine the precise impact of specific venom components on patient outcomes. Future studies should prioritize the collection of detailed information on snake species involved in each case, enabling a more targeted analysis of prognostic factors.

## 5 CONCLUSION

This study analyzed snakebite envenomation in northern Iran, focusing on four venomous snake species. Most cases occurred in males in their early thirties during summer, with upper extremities being commonly affected. Edema, ecchymosis, and blisters were common local signs, while gastrointestinal disturbances were the main systemic complication. The Snakebite Severity Score (SSS) correlated with ICU admission, poor treatment response, and longer hospital stays. Specific antivenom administration and supportive care played crucial roles in patient management, with no reported mortality. Further research with larger samples and accurate species identification is needed to optimize treatment strategies and improve outcomes.

## AUTHOR CONTRIBUTIONS

**Hoorvash Farajidana**: Conceptualization; Writing—review and editing. **Seyedarad Mosalamiaghili**: Writing—review and editing; Writing—original draft; Formal analysis; Methodology; Software. **Kasra Assadian**: Formal analysis; Writing—original draft; Writing—review and editing; Visualization. **Soodeh Jahangiri**: Formal analysis; Writing—review and editing; Writing—original draft. **Maryam Masumzadegan**: Conceptualization; Data curation; Methodology. **Farangis Sadeghi**: Conceptualization; Methodology; Investigation; Supervision. **Lida shojaei Arani**: Supervision; Resources; Conceptualization; Data curation; Methodology; Project administration.

## ETHICS STATEMENT

Informed consent was obtained from all patients; the ethics committee of the Alborz University of Medical Sciences approved this study (Approval number: IR.ABZUMS.REC.1401.135).

## TRANSPARENCY STATEMENT

The lead author Lida shojaei Arani affirms that this manuscript is an honest, accurate, and transparent account of the study being reported; that no important aspects of the study have been omitted; and that any discrepancies from the study as planned (and, if relevant, registered) have been explained.

## Data Availability

The data that support the findings of this study are available on request from the corresponding author. The data are not publicly available due to privacy or ethical restrictions. The datasets used and/or analyzed during the current study are available from the corresponding author upon reasonable request.
